# Testing gene by community disadvantage moderation of sexual health outcomes among urban women

**DOI:** 10.1371/journal.pone.0223311

**Published:** 2019-10-03

**Authors:** Terrinieka W. Powell, Jill A. Rabinowitz, Michelle R. Kaufman, Adam J. Milam, Kelly Benke, Danielle Y. Sisto, George Uhl, Brion S. Maher, Nicholas S. Ialongo

**Affiliations:** 1 Department of Mental Health, Johns Hopkins Bloomberg School of Public Health, Baltimore, MD, United States of America; 2 Department of Population, Family and Reproductive Health, Johns Hopkins Bloomberg School of Public Health, Baltimore, MD, United States of America; 3 Department of Health, Behavior and Society, Johns Hopkins Bloomberg School of Public Health, Baltimore, MD, United States of America; 4 New Mexico VA HealthCare System, Las Vegas, NM, United States of America; Boys Town National Research Hospital, UNITED STATES

## Abstract

We examined whether the interplay between community disadvantage and a conduct disorder polygenic risk score (CD PRS) was associated with sexual health outcomes among urban women. Participants (*N* = 511; 75.5% African American) were originally recruited to participate in a school-based intervention and were followed into adulthood. Community disadvantage was calculated using census data when participants were in first grade. At age 20, blood or saliva samples were collected and participants reported on their condom use, sexual partners, and sexually transmitted infections. A CD PRS was created based on a genome-wide association study conducted by Dick et al. [2010]. Higher levels of community disadvantage was associated with greater sexually transmitted infections among women with a higher CD PRS. Implications of the study findings are discussed.

## Introduction

Youth are more likely than adults to engage in risky sexual behaviors, including sex without condoms, sex with multiple partners, and substance use in conjunction with sex [1]. These risky behaviors can result in negative consequences including teen pregnancy, reduced educational attainment, and unemployment [2]. Young women residing in disadvantaged neighborhoods are disproportionately at risk for poor sexual health outcomes [3–5]. For example, young women living in socio-economically deprived communities are more likely to use condoms inconsistently and contract sexually transmitted infections (STIs) than those in communities with greater resources [3,6,7]. In resource-poor contexts, women may experience higher levels of poverty and have limited access to reproductive health care services, thus increasing the likelihood that they will have fewer opportunities to engage in consistent safer sex practices [7,8]. However, women living in disadvantaged communities likely display diversity in their sexual risk taking. The factors that contribute to differences in sexual behaviors may be related to individual specific-factors (i.e., genetic variants) that predispose some women towards harmful sexual behaviors and their consequences. Identifying these genetic variants may elucidate factors that are associated with heterogeneity in sexual risk taking.

### Links between conduct disorder and sexual health outcomes

Conduct disorder (CD) is defined as a syndrome characterized by a pattern of antisocial behaviors that often infringes on the rights of others and violates social norms [9,10]. CD has been consistently associated with sexual health outcomes among women. For example, women higher in CD phenotypes (e.g., impulsivity, disobedience) are more likely to engage in earlier sexual debut, use contraceptives less frequently, have more sexual partners and STIs, and experience early child-rearing relative to women lower in CD phenotypes [10–12]. Individuals higher in CD symptoms may be higher in impulsivity, sensation seeking, and may be more likely to seek out immediate rewards despite potential negative repercussions [9,13], all of which may predispose these individuals towards sexual risk taking.

In studies examining the relationship between CD symptoms and sexual health outcomes, CD symptoms are typically examined phenotypically, or via observations of CD emotional and behavioral characteristics. Genetic variants associated with CD symptoms may also play a role in sexual risk behaviors and may be particularly relevant to consider among women in young adulthood, though research is lacking. Contextual changes (e.g., employment, establishing a residence) and increased independence that are associated with young adulthood may enable women to select environments that allow for the expression of their genetic predispositions [14]. Advances in genomics and the genetics of CD and related traits offer the possibility of clarifying genetic variants associated with sexual health outcomes and may inform prevention and intervention initiatives aimed at reducing these outcomes among women residing in underserved communities. Consistent with relations identified between phenotypic conduct problems and sexual health outcomes [15], higher genetic load for CD may be associated with sexual risk behaviors, though it is unclear whether this is the case based on prior work.

### Contextual factors related to sexual health outcomes

Although higher genetic loading for CD may predispose young women towards risky sexual behaviors, it is likely that this relationship depends on environmental influences. Consistent with bioecological models, the interaction between individuals’ characteristics and distal contextual factors, such as community disadvantage, may influence risk for problem behaviors [16]. Higher levels of community disadvantage are associated with less condom use and a greater number of sexual partners among young people [17–19]. Individuals who live in communities characterized by higher poverty and limited institutional resources may have reduced access to sexual and reproductive health care [17]. Moreover, young women residing in disadvantaged communities may internalize neighborhood norms (e.g., acceptability of teenage pregnancy), which may increase the likelihood of these women engaging in sexual risk behaviors [20]. Limited work has indicated that exposure to familial disadvantage (i.e., low socioeconomic status) is associated with an increased risk for teenage pregnancy among young women with higher levels of conduct problems [10]. However, it is unclear whether these findings would apply when considering genetic influences of CD, community disadvantage, and other indices of sexual risk taking.

While phenotypic CD symptoms and community disadvantage have been shown to be independently predictive of sexual health outcomes in several studies and our sample [15,21], no studies to our knowledge have examined whether sexual behaviors are associated with (a) genetic influences underpinning CD and/or (b) the interaction of these genetic influences with community disadvantage. The present study sought to address these gaps by examining genetic load for CD, as well as the interactive effects of CD genetic load and contextual factors on sexual risk behaviors.

## Material and methods

### Participants

The analytic sample was drawn from two cohorts of participants in a mid-Atlantic region of the U.S. as part of a series of randomized controlled trials of elementary school-based universal prevention interventions. The interventions were implemented in first grade with the goals of improving academic achievement and reducing aggressive/disruptive behaviors. The participants were followed periodically from first grade to young adulthood, with 2,311 individuals available for recruitment in first grade. The study took place beginning in 1985 and ended in 2011 and was approved by a University Institutional Review Board. Participants provided informant consent as adults and assent as youth [22].

The analytic sample included 511 women (75.5% African Americans) for whom genome-wide assays using Affymetrix 6.0 SNP microarrays were successfully completed in DNA samples obtained at approximately age 20 [23]. The school district provided information on the students’ sex, race, and free/reduced priced meal status (a proxy for income described in more detail below) in first grade. Participant demographics for the analytic sample are outlined in Table 1. In terms of differences between the analytic sample and those not assessed at approximately age 20 and/or those who did not provide DNA, the analytic sample had a significantly (*p* < .05) greater number of participants who (a) qualified for free/reduced priced meals (analytic sample, 72.6% vs. whole sample, 64.5%); and (b) were African American (analytic sample, 73.5% vs. whole sample, 60.8%). No significant differences were found between the analytic sample and the whole sample in terms of intervention status.

**Table 1:** Sample characteristics.

Characteristic	*n* (%)
Race	
African American	386 (75.5%)
European American	125 (24.5%)
Free/reduced meal status	
Yes	371 (72.6%)
No	139 (27.2%)
Intervention	
Yes	211 (41.3%)
No	300 (58.7%)
Education^a^	
<High school	218 (42.7%)
High school or GED	170 (33.3%)
Vocational training/college	121 (23.7%)
Income^a^	
<$10,000	202 (47.2%)
$10,000 - $20,000	125 (24.5%)
>$20,000	46 (8.8%)
Cohort identification	
Cohort 1	281 (55.0%)
Cohort 2	230 (45.0%)

^a^Education and income information were obtained from participants at age 20.

*Note*. GED = General Education Degree.

### Variables

#### Community Disadvantage

We geocoded all available participants’ home addresses when participants were in first grade. A community disadvantage score was calculated using census-tract level items from the 1990 and 2000 Decennial census [24]. The items used to create the index included the percentages of (a) adults 25 years or older with a college degree, (b) owner-occupied housing, (c) households with incomes below the federal poverty threshold, and (d) female-headed households with children. The following formula was used to calculate the community disadvantage score: {[(c / 10 + d / 10)–(a / 10 + b / 10)] / 4}[25]. A one-unit increase in the community disadvantage score is equivalent to an increase of 10 percentage points for each component item of the index. Higher scores reflect higher levels of disadvantage.

#### DNA and genotyping

Blood or buccal samples were obtained in young adulthood with 84.1% of the study sample providing a blood sample and the remainder providing a saliva sample. DNA was extracted and genotyped using Affymetrix 6.0 microarrays according to manufacturer’s instructions [23]. These chips allow genotyping of approximately 1 million SNPs across the genome. Inclusion in the analysis required that each sample pass Affymetrix quality-control procedures [26]. A small number of samples collected initially failed to meet quality-control standards. When this occurred, new samples were collected from the participants and the assays were rerun. Genotype calling was done using Affymetrix Power Tools. A series of quality control steps were performed at the individual and SNP level. Subjects with >5% missing genotype data were removed. SNPs were removed from further analysis when they had a minor allele frequency < .01, missingness > 0.05, or departures from Hardy–Weinberg equilibrium at *p* < .0001. These steps were performed using PLINK 2.0 [27].

#### Polygenic risk score for conduct disorder

A CD PRS was created based on a genome-wide assay to identify genetic factors associated with CD symptomatology [28]. The authors conducted a genome wide association study (GWAS) for CD symptomatology, which included participants who met diagnostic criteria for alcohol dependence from the Study of Addiction: Genes and Environment (SAGE) [28]. The CD discovery GWAS included a diverse ancestry sample (*N* = 2698 European ancestry; *N* = 1257 African ancestry) of adults that retrospectively reported on their CD symptoms. In our sample, the CD PRS has been positively correlated with childhood aggression [29].

#### Population stratification

It is important to identify and control for population stratification, or genetic differences between subpopulations so that any significant associations found are not confounded by ancestry [30]. Principal components analysis in PLINK was used to create population stratification control variables. This process uses an orthogonal transformation to extract principal components (PCs) from genome-wide SNP variables. The first principal component explains as much of the variance in the data as possible. Subsequent principal components explain as much of the remaining variance as possible without being correlated with previous components. In total, ten principal components were identified. Top single nucleotide polymorphisms (SNPs) (nominal *p* < 0.001) were included in the polygenic score. The raw CD polygenic score was regressed on the ten principal components that we identified. The standardized residuals from these regressions are the continuous ancestry-adjusted (or corrected) scores used in the primary analyses.

#### Sexual health outcomes

Number of sexual partners, frequency of condom use, and STIs were assessed using a questionnaire developed by the AIDS Linked to the Intravenous Experience (ALIVE) study [31]. About 10% percent of participants were interviewed in person, while approximately 90% of participants were interviewed over the phone by trained research assistants. To assess number of partners, participants were asked, “About how many partners have you had vaginal sex with since the first person?” Frequency of condom use was assessed using the item, “How often did you use a condom with your partners during vaginal sex?” which was rated on a 6-point Likert scale (1 = *always* to 6 = *never*). Sixteen questions also asked participants to report their lifetime history of STIs. Sample items are “How many times have you been told by a doctor or health professional that you had gonorrhea?” and “How many times have you been told by a doctor or health professional that you had herpes?”

## Statistical analyses

Pearson’s correlations were conducted to investigate the relationships among the continuous variables, point biserial correlations were used to investigate relations among categorical and continuous variables, and Cramer’s phi was used to estimate the correlations between categorical variables using SPSS Version 25 [32]. The primary analyses were conducted using Mplus Version 8.0 [33]. The demographic and participant variables were coded as follows: (European-American (Caucasian) = 0, African American = 1; no intervention = 0, received an intervention = 1; no free/reduced priced meals = 0, received free/reduced priced meals = 1). All continuous predictor variables were standardized. Preliminary analyses were conducted to test for gene by environment correlations and for distributions of these data. Significant correlations between genetic and environmental factors may indicate that genes and environments are not independent of each other and can result in spurious interaction effects [34]. Thus, we tested for this possibility.

Owing to the count nature of the dependent variables (i.e., number of sexual partners and STIs), poisson regressions were the primary analytic technique employed. Preliminary analyses indicated overdispersion in the distribution of number of STIs and sexual partners as the variances exceeded their means and thus, negative binomial regressions were used to conduct analyses involving these variables. Unlike other methods for analyzing count data, negative binomial regressions include an extra parameter that account for overdispersion [35]. For frequency of condom use, a linear regression was conducted.

We examined whether community disadvantage moderated the relationship between the CD PRS and sexual behaviors. Each of the regressions controlled for participant intervention status, the CD PRS, community disadvantage, and free/reduced priced meal status; this variable is often considered a proxy for family income [36,37] and has been robustly associated with a number of negative outcomes among youth [38,39]. Although individuals and families with low incomes may be more likely to live in disadvantaged communities, there still may be variation in family incomes in these neighborhoods. Thus, we controlled for free/reduced priced meal status to ensure that our results were driven by the neighborhood context and not family income.

The significance of interactions was evaluated using a false discovery rate [40] of 0.05.

For interactions that were at or below this threshold, simple slopes were computed to reflect higher, average, and lower community disadvantage that were at the mean and ± 1 *SD* from the mean of community disadvantage, respectively, consistent with the moderation approaches outlined by Aiken & West [41] and Holmbeck [42]. We tested whether different levels of the moderator (high, average, and low community disadvantage) and the outcomes were significantly different from zero and dependent on the independent variable (the CD PRS). Post hoc regressions involved entry of the predictors (e.g., the CD PRS variable), the community disadvantage (at the mean and ± 1 *SD*) variable, and the CD PRS × community disadvantage interaction. We graphed the slopes and intercepts in regression equations that were at the mean ± 1 *SD* from the mean of community disadvantage.

## Results

Descriptive statistics of the study variables are highlighted in Table 2. Correlations among study variables are presented in Table 3. There was not a significant correlation observed between free/reduced priced meal status and intervention status, φ = .039, *p* = .672. The CD PRS was not significantly correlated with number of sexual partners, number of STIs, or condom use frequency. There was a small, non-significant negative correlation between the CD PRS and community disadvantage. Given these non-significant associations, we were able to rule out gene and environment correlations.

**Table 2:** Descriptive statistics for study variables.

	*n*	*M*	*SD*	Range
CD PRS^a^	511	0.002	0.95	-2.74–2.77
Disadvantage	498	-0.52	1.25	-2.77–1.76
STIs^b^	497	0.93	1.19	0–7
Number of partners	466	4.74	6.24	0–63
Condom use	434	4.77	1.45	1–9

^a^CD PRS = conduct disorder polygenic risk score.

^b^STIs = sexually transmitted infections.

**Table 3:** Bivariate correlations among study variables.

*Variable*	*1*	*2*	*3*	*4*	*5*	*6*	*7*
1. Free/reduced priced meal status^a^	--						
2. Intervention status^a^	--	--					
3. CD PRS^b^	.004 (.933)	.01 (.785)	--				
4. Disadvantage	.51 (.000)	.08 (.066)	.02 (.644)	--			
5. Sexual partners	.01 (.754)	.02 (.719)	.07 (.163)	-.0004 (.994)	--		
6. Condom use	.10 (.035)	-.11 (.036)	.002 (.971)	.07 (.152)	-.04 (.418)	--	
7. STIs^c^	.18 (.000)	.07 (.150)	.11 (.017)	.21 (.000)	.41 (.000)	-.09 (.064)	--

^a^Correlations between categorical variables are presented in the text.

^b^CD PRS = conduct disorder polygenic risk score.

^c^STIs = sexually transmitted infections.

Note. *p*-values are presented in parentheses.

The CD PRS × community disadvantage interaction did not predict number of partners (OR = 0.99, 95% CI = 0.88–1.11, *p* = 0.892) or condom use (*B* = 0.07, *p* = 0.319) (Table 4). The CD PRS was not associated with condom use (*B* = -0.001, *p* = .994); however, the CD PRS showed a positive association with number of sexual partners (OR = 1.11, 95% CI = 1.00–1.23, *p* = 0.063).

**Table 4:** Summary of analyses predicting total number of STIs, number of partners, and frequency of condom use from the interplay between community disadvantage and the CD PRS.

	**OR (95% CI)**		***p-*value**
**Number of STIs**^a^			
Intervention status	0.96 (0.77–1.20)		.745
Free/reduced priced meal status	1.47 (1.10–1.96)		.010
CD PRS^b^	1.08 (0.97–1.20)		.185
Community disadvantage	1.20 (1.06–1.35)		.003
CD PRS × community disadvantage	1.14 (1.03–1.27)		.011
**Number of sexual partners**			
Intervention status	1.04 (0.81–1.33)		.774
Free/reduced priced meal status	1.04 (0.78–1.39)		.785
CD PRS	1.11 (0.99–1.24)		.070
Community disadvantage	0.99 (0.86–1.14)		.895
CD PRS × community disadvantage	0.99 (0.88–1.11)		.892
	***B* (SE)**	**β**	***p*-value**
**Frequency of condom use**			
Intervention status	-0.10 (.14)	-0.03	.483
Free/reduced priced meal status	0.29 (.19)	0.09	.122
CD PRS	-0.02 (.08)	-0.01	.775
Community disadvantage	0.05 (.08)	0.03	.571
CD PRS × community disadvantage	0.07 (.07)	0.05	.319

^a^STIs = sexually transmitted infections.

^b^CD PRS = conduct disorder polygenic risk score.

Note. OR = odds ratio; CI = confidence interval.

**Fig 1:**
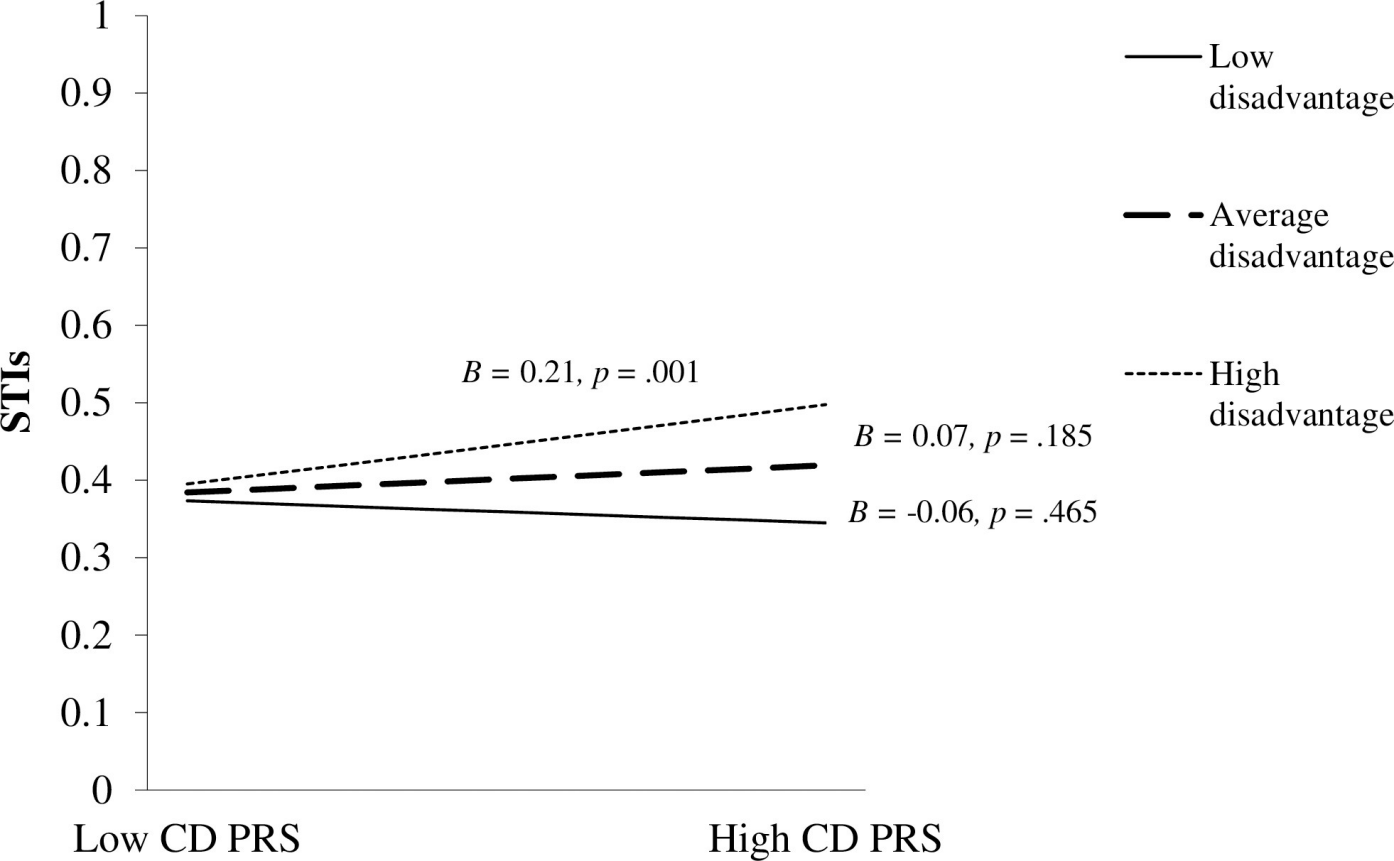
Relation between the conduct disorder polygenic risk score (CD PRS) and sexually transmitted infections (STIs) in the context of different levels of community disadvantage (plotted at the mean and +/- 1 SD from the mean).

The CD PRS × community disadvantage interaction was associated with a higher incidence of STIs (OR = 1.14, 95% CI = 1.03–1.27, *p* = 0.011) (Table 4), and remained significant after correcting for multiple testing (Benjamini-Hochberg adjusted *p*-value = .033). Post hoc probing indicated that the slope for high (*B* = 0.21, *p* = 0.001) community disadvantage was significant (Fig 1). However, the slopes for average (*B* = 0.07, *p* = 0.185) and low community disadvantage (*B* = -0.06, *p* = 0.465) were not significant. In other words, females with a higher CD PRS had a greater likelihood of reporting STIs when exposed to higher community disadvantage, but not average or lower levels of community disadvantage.

## Discussion

This is the first study, to our knowledge, to examine whether the interaction between genetic loading for CD and community disadvantage is associated with sexual health outcomes among urban women. Past work has indicated that behavioral reports of conduct problems and community disadvantage are individually predictive of sexual risk taking [9,20]. Results from the present study extend previous findings by highlighting the combined effects of genetic propensity for CD and disadvantage on sexual risk behaviors among women. In particular, exposure to higher levels of community disadvantage among women with greater genetic load for CD increases their risk for STIs.

A higher genetic load for CD was positively associated with number of sexual partners. These findings are consistent with previous work indicating that women higher in CD phenotypes (e.g., defiance) are more likely to have a greater number of sexual partners [10–12]. Our findings may reflect the propensity of young women with higher CD genetic loading to seek out novel experiences, such as having multiple sexual partners, given that these women may be more thrill-seeking. Women with higher CD genetic loading may also have multiple sexual partners as a form of rule-breaking given conventional gender norms that encourage women to have fewer sexual partners relative to men [43]. Moreover, it is also feasible that women with a higher CD PRS may affiliate with deviant peers who are more likely to engage in riskier behaviors (i.e., substance use during sex) and pursue high-risk settings (e.g., clubs) where the opportunity to meet multiple partners is present.

A higher genetic load for CD was not associated with frequency of condom use. These findings conflict with previously-reported links between elevated phenotypic conduct problems and decreased contraceptive use [11]. The use of condoms may be influenced by a variety of factors such as condom self-efficacy, relationship characteristics, and partner’s preference [44, 45]. These factors may better account for consistency in condom use than women’s genetic load for CD. Future research should examine the mechanisms through which polygenic load for CD is associated with number of partners, but not frequency of condom use.

A greater genetic propensity for CD interacted with community disadvantage to predict number of STIs. In disadvantaged communities characterized by higher levels of socioeconomic deprivation, women may have limited access to medical care [17]. Coupled with a higher genetic load for CD, limited access to safe and affordable sexual and reproductive health services may decrease women’s opportunities to seek out prevention and treatment services for medical conditions [46]. This context may be particularly harmful among women with a higher CD PRS given their potential propensity for risk taking and disregard for social norms that may set them on a trajectory towards behaviors that exacerbate their risk for STIs. Thus, higher levels of community disadvantage may act as a vulnerability for compromised sexual and reproductive health among these women.

There are limitations to this study. The CD PRS was derived from a GWAS with a relatively small sample size (<4,000 adults) [28]. Research has shown that the predictive ability of PRS generally increases with larger discovery samples and when the ancestry of the discovery sample matches that of the target sample [30,47]. Indeed, smaller discovery samples or a mismatch between the ancestry of the target and discovery sample may result in an attenuation in the variance accounted for and directional inconsistencies between the PRS and phenotypes under study [48].While the CD GWAS discovery sample is smaller relative to other GWASs on externalizing behavior (e.g., [49,50]), the CD GWAS includes about a third of individuals of African ancestry, potentially increasing the predictive utility of this score in our sample, a predominantly African ancestry sample. Nevertheless, replication of our findings are necessary using larger, admixed samples and other polygenic risk scores to validate our study findings, ideally scores that are derived from GWASs of mixed-ancestry or predominantly African ancestry populations. Differences in allele frequencies, linkage disequilibrium, and population history further highlights the need for additional gene identification efforts among diverse ancestry populations.

These limitations notwithstanding, this study makes a number of contributions to the literature and has several important implications. The present study employed a prospective, longitudinal design, allowing us to draw conclusions regarding the effect of early community disadvantage with individual factors in predicting sexual risk taking in adulthood among urban women. Our findings suggest that the combination of genetic propensity for CD and the experience of early community disadvantage may influence liability to compromised sexual and reproductive health. Before such findings could be used to inform intervention efforts, large scale replication of our findings are needed, in addition to incorporation of underrepresented, diverse ancestry populations in GWASs, societal acceptance of genetic screening for behavioral outcomes, as well as advances in translational research regarding how to implement gene by environment findings such as ours into prevention programming.
